# *The Organon* and Materia Medica Club of the Bay Cities of California

**Published:** 1897-02

**Authors:** W. E. Ledyard

**Affiliations:** Secretary


### *THE ORGANON* AND MATERIA MEDICA CLUB OF THE BAY CITIES OF CALIFORNIA.

The regular semi-monthly meeting was held at the office of Dr. George H. Martin, 606 Sutter Street, San Francisco, Friday evening, July 3d, 1896.

Members present: Drs. J. M. Selfridge, A. McNeil, M. T. Wilson, G. J. Augur, W. E. Ledyard, C. M. Selfridge, M. F. Underwood, and George H. Martin.

The meeting was called to order at 8.30 o'clock by the President, Dr. J. M. Selfridge. The minutes of the previous meeting were read by the Secretary, and after corrections by Drs. J. M. Selfridge and Augur, were approved.

Dr. McNeil presented an amendment to the Constitution providing for senior and junior membership. On motion of Dr. Martin, seconded by Dr. Wilson, the amendment was unanimously adopted.

Dr. J. M. Selfridge commenced the reading of *The Organon*, at Section 160.

Dr. Augur—A young man from Stanford University came to me some time ago suffering with a skin disease which he had contracted from sleeping in a bed that had been occupied by another young man with the same disease. My patient had been suffering a month or two with the trouble. I gave him one dose of *Sulphur*$^{200}$. Two weeks later I received a letter from him stating that he was much worse; that the itching was driving him crazy; the skin was peeling off in layers, fine, white, powdery scales, the tissues underneath being red, raw, and angry, particularly around the scrotum. I sent him one dose of *Psorinum*$^{50,000}$. He received benefit very soon, and in ten days was cured.

Sections 161–168.

Dr. McNeil—The fact that in the course of six, eight or ten days after the administration of the medicine in chronic diseases an aggravation occasionally occurs, shows the importance of not repeating the dose too frequently. We should give one or two doses and then wait for the action. It is striking to see here how Hahnemann with the few remedies he had could do such good work. We have had many more remedies without doing much more than he did with the few. I confess that I have never been able to cure in the manner which he delineates here.

Sections 169–171.

### Discussion.

Dr. McNeil—The habit of alternating remedies is utterly without excuse.

Dr. Augur—It frequently happens that we might have to use two or three remedies, but not in the manner that we understand as alternation.

Dr. McNeil—There is one point that should be remembered when changing from one remedy to another, and that is whether the second remedy will follow the first one well or not. We have inimical and complementary remedies, and this fact should not be forgotten when giving the second remedy.

Dr. J. M. Selfridge—The second remedy should not be given simply because it will follow the first one well, but a new picture of the case must be taken which must correspond to the characteristic symptoms of the new remedy.

Dr. McNeil—An article written by Bœnninghausen, I translated for the International Hahnemannian Association in 1893, in which he lays it down as a general rule that a drug should never follow itself. In cases where the drug has *relieved*, without *removing* any of the symptoms, the drug should be repeated.

Dr. J. M. Selfridge—Do we understand that if you are treating a chronic disease, and the patient is relieved, that you are not to repeat the dose?

Dr. McNeil—We would repeat the dose if the symptoms all remained, but were somewhat relieved. If the drug entirely removes any of the symptoms, a new picture is presented which calls for a new drug.

Dr. Ledyard—This strikes me as being remarkably important. If one or more symptoms are cured, the picture of the case cannot be as it was at first.

Dr. Underwood—The testimony of modern homœopaths would hardly bear out this statement of Bœnninghausen's. I will relate a case for illustration: A case of mine in which *Sulphur* was the remedy. I gave one dose, and in a week every symptom had disappeared, but other symptoms came up. I checked the case up and found no drug to finish the cure. I looked up *Sulphur*, and it completely covered the case again. I did this a third time, gave *Sulphur* and cured.

Dr. McNeil—In Indiana, during the winters of 1873 and 1874, the epidemic remedy was *Mercurius*. In every acute disease the patient was sweating, a warm, profuse sweat. In one case of pneumonia the patient was literally steaming. I gave *Mercurius* and cured. The next winter *Mercurius* was the epidemic remedy again, but the patients were not sweating. The salivary glands seemed to be more in action this winter. In looking up a case for a new remedy and we find that the old remedy still covers it, it should be given.

Dr. Augur—If a symptom disappears while giving the first remedy may it not come back again? A young lady patient of mine had suffered with indigestion for years. I gave her *Pulsatilla* at intervals of six weeks for three months. At times she would be absolutely free from symptoms, and then they would return again. I think if I had changed the remedy in this case I would not have had the good results that I did have.

The meeting was then declared adjourned, to meet again the third Friday in July at Dr. Martin's office, when the reading of *The Organon* would be commenced at Section 172.

W. E. Ledyard, B. A., M. B., etc.,
*Secretary.*

Reported by Eleanor F. Martin, M. D.

---

